# Supplementary material for: Hybrid Models and Biological Model Reduction with PyDSTool
Source: PLoS Comput Biol. 2012 Aug 9;8(8):e1002628. doi: 10.1371/journal.pcbi.1002628 (PMC3415397; doi:10.1371/journal.pcbi.1002628)
Supplement: Text S4 — Complete source code for the PyDSTool package (version 0.88.120504). Includes API documentation and help files linking to web pages. This file is identical to the current public release on Sourceforge.net. (ZIP) [file pcbi.1002628.s004.zip › PyDSTool/html/PyDSTool.Toolbox.optimizers.defaults-module.html]

xml version="1.0" encoding="ascii"?


PyDSTool.Toolbox.optimizers.defaults


| Home | Trees | Indices | Help | PyDSTool |
| --- | --- | --- | --- | --- |

Package PyDSTool ::
Package Toolbox ::
Package optimizers ::
Module defaults

[hide private]

[frames] | no frames]

# Module defaults

source code

Defines the defaults parameters for the generic optimizer
framework


|  |  |
| --- | --- |
| Variables | [hide private] |

SMALL\_DF = `2`

SMALL\_DELTA\_X = `3`

SMALL\_DELTA\_F = `4`

SMALL\_DELTA\_X\_X = `5`

SMALL\_DELTA\_F\_F = `6`

FVAL\_IS\_ENOUGH = `10`

SOLVED\_WITH\_UNIMPLEMENTED\_OR\_UNKNOWN\_REASON = `1000`

IS\_NAN\_IN\_X = `-4`

IS\_LINE\_SEARCH\_FAILED = `-5`

IS\_MAX\_ITER\_REACHED = `-7`

IS\_MAX\_CPU\_TIME\_REACHED = `-8`

IS\_MAX\_TIME\_REACHED = `-9`

IS\_MAX\_FUN\_EVALS\_REACHED = `-10`

IS\_ALL\_VARS\_FIXED = `-11`

FAILED\_WITH\_UNIMPLEMENTED\_OR\_UNKNOWN\_REASON = `-1000`

parameters = `{'alpha_step': 1.0, 'ftol': 0.001, 'gtol': 0.001,...`

errors = `{-1000: 'Unknown reason of failure', 2: 'gradient nor...`


|  |  |
| --- | --- |
| Variables Details | [hide private] |

### parameters

Value:
:   ```
    {'alpha_step': 1.0,
     'ftol': 0.001,
     'gtol': 0.001,
     'iterations_max': 1000,
     'min_alpha_step': 1.0,
     'xtol': 0.001}
    ```

### errors

Value:
:   ```
    {-1000: 'Unknown reason of failure',
     2: 'gradient norm is small enough',
     3: 'absolute X difference is small enough',
     4: 'absolute F(X) difference is small enough',
     5: 'relative X difference is small enough',
     6: 'relative F(X) difference is small enough',
     10: 'F(X) is small enough',
     1000: 'Unknown reason of convergence'}
    ```

  


| Home | Trees | Indices | Help | PyDSTool |
| --- | --- | --- | --- | --- |

|  |  |
| --- | --- |
| Generated by Epydoc 3.0.1 on Fri May 4 15:24:05 2012 | http://epydoc.sourceforge.net |
